# An Update on Vaccines Against *Trypanosoma cruzi* and Chagas Disease

**DOI:** 10.3390/pathogens14020124

**Published:** 2025-01-30

**Authors:** Nisha J. Garg

**Affiliations:** 1 Department of Microbiology & Immunology, University of Texas Medical Branch (UTMB), Galveston, TX 77555-1070, USA; nigarg@utmb.edu; 2 Institute for Human Infections and Immunity, University of Texas Medical Branch (UTMB), Galveston, TX 77555-1070, USA

**Keywords:** Chagas, *Trypanosoma cruzi*, vaccine

## Abstract

Chagas disease (CD) is a global health concern, with no existing therapies to prophylactically treat adults traveling to endemic countries or those who may already be infected with *Trypanosoma cruzi*. The economic burden of Chagas cardiomyopathy and heart failure, due to healthcare costs and lost productivity from premature deaths, provides a strong rationale for investment in the development of immune therapies against CD. Vaccine efficacy is proposed to depend heavily on the induction of a robust Th1 response for the clearance of intracellular pathogens like *T. cruzi*. In this review, updated information on the efforts for vaccine development against CD is provided.

## 1. Introduction

*Trypanosoma cruzi (T. cruzi* or *Tc*) is a kinetoplastid parasite that is the causative agent of Chagas disease (CD). *T. cruzi* is endemic in the Americas, ranging from southern United States to Argentina, due to continuous transmission between triatomine vectors and wildlife and domestic reservoir hosts. Acute blood parasitemia and non-specific somatic symptoms resolve into the subclinical (indeterminate) form of disease within 2–4 months after infection. Decades later, infected individuals may advance to the chronic (determinate) form of CD, involving cardiac, gastrointestinal, and neurological complications [1]. Though under-reported and under-estimated, CD affects 6–8 million people and results in up to 12,000 deaths annually [2]. Approximately 71 million people are exposed to risk of infection and ~28,000 new cases of *T. cruzi* infection occur every year [3]. Recent data suggest that CD results in the loss of 0.7 million disability-adjusted life years [4] and an economic burden of >USD 10 billion [5] per year on the American continent. Even after applying stringent simulation parameters of a 1% infection risk and treatment efficacy of 25%, computational modeling studies suggest that a vaccine against *T. cruzi* costing USD 20/dose would be economically viable and provide net cost savings [5,6]. With an efficacy of >50% and infection risk of 20%, a vaccine costing even >USD 200 per dose is estimated to be economically advantageous [5]. Furthermore, a safe, effective, and reliable vaccine would provide relief from the costs and efforts associated with vector control to reduce parasite dissemination, and the diagnosis and treatment of individuals that are infected in endemic countries.

Upon infection, *T. cruzi* employs a variety of strategies to evade or suppress immune detection and disseminate through the bloodstream to establish active invasion and intracellular replication in a variety of host tissues [7,8]. The host immune system still does a great job in controlling the acute parasitemia, but low-grade tissue infection persists and provides for consistent activation of inflammatory reactions and CD pathogenesis [1]. Several questions as to how parasites persist at low levels remain unanswered: Is it that a few parasites escape from the immune system by changing the dominant antigens on their surface as is noted in *T. brucei*, or does the parasite adopt a different life-cycle stage that is not noted in in vitro cultures and acute in vivo infection? Some studies have suggested that the parasite persists in the gastro-intestinal tract [9] and adipose tissue [10,11] to support a chronic inflammatory state. At least one study documented that a latent form of *T. cruzi* amastigotes, such as that of *Mycobacterium tuberculosis* and *Toxoplasma gondii*, escaped from immune detection and served as so-called persisters [12], though how it differentiated into persister form was not studied. Nevertheless, the finding of low levels of circulating parasites in chronically infected individuals provides strong evidence that *T. cruzi* does come out of dormancy and hiding to maintain an active intracellular/infective life cycle, at least intermittently. These studies provide a basis for recommending drug and immune therapies for parasite control in the indeterminate stage of CD.

Pathogenesis of chronic Chagas disease is attributed to inflammatory immune-mediated myocardial injury, autonomic nervous system derangements, and microvascular disturbances, and has been discussed in several reviews [1,13]. Furthermore, it had been documented that mitochondrial dysfunction of the respiratory chain results in increased generation of reactive oxygen species (ROS) that predispose the heart to oxidative insult during *Tc* infection and chronic disease [14,15]. The sustained occurrence of oxidative adducts was noted in the myocardium of experimental models of CD and in the peripheral blood of rodents and human Chagas subjects [16,17,18], which was exacerbated by inefficient antioxidant capacity [19,20]. Other studies demonstrating ROS signaling of cytokines and chemokines production in infected cardiomyocytes and murine hearts have provided a potential mechanistic link between ROS generation and chronic inflammation in Chagas cardiomyopathy [15,21]. Readers interested in further understanding the complexity of Chagas disease pathogenesis and efforts toward vaccines, therapies, and drugs development are also referred to excellent recent reviews [22,23,24,25].

## 2. Immunity to *T. cruzi* Infection

The innate immune system is the first to actively elicit a defensive role, followed by the adaptive immune response. It is well documented that *T. cruzi* evades complement pathways to survive in the mammalian host and establish persistent infection [26]. *T. cruzi* surface glycoproteins (mucins), glycophospholipids, and other molecules stimulate the production of cytokines (e.g., IFNγ, TNFα, IL1β, IL6), chemokines (e.g., MCP1, RANTES/CCL5, IP10), and free radicals (e.g., superoxide, nitric oxide, peroxynitrite) in macrophages and cardiomyocytes [27,28,29]; however, these responses are either delayed or occur at sub-par level and fail to clear the parasite [30,31]. Studies in animal models of *T. cruzi* infection suggest that release of IFNγ and IL12 by natural killer cells and macrophages is essential for the stimulation of adaptive type 1 T cell response [32,33]. CD4^+^T cells produce Th1 cytokines (IFNγ, IL2) and assist in parasite control through amplification of the phagocytic activity of macrophages, and stimulation of B cells and CD8^+^ T cells [34,35]. Antibodies produced by B cells must promote opsonization, phagocytosis, and complement-mediated killing of the parasite; and antibodies to the Galα(1,3)Galβ(1,4)GlcNAc epitope of surface expressed mucin glycoproteins exhibit such anti-parasite activity [36]. Finally, *Tc*-specific CD8^+^T cells, detected in infected mice and humans, contribute to *Tc* control by cytolysis of infected cells and secretion of Th1 cytokines that induce trypanocidal activity [37].

Immunological studies in chronically infected mice and patients have yielded conflicting results. Some reports indicated that immune exhaustion of CD4^+^ and CD8^+^ T cells contributes to parasite persistence [38], while others concluded that excessive production of proinflammatory cytokines correlates with tissue damage and clinical disease in chronically infected patients [39]. Recently, it was proposed that antigen-presenting cells producing IL27 contribute to a balanced proinflammatory/anti-inflammatory (IFNγ/IL10) response, leading to reduced inflammatory infiltrate in the myocardium of chagasic mice [40]. Overall, it is safe to conclude from the current literature that an efficient protective response to *T. cruzi* infection requires the combined activities of phagocytes, T helper cells, and cytotoxic T lymphocytes capable of rapidly killing the circulating and intracellular parasites. A sub-par activation of any of these components can result in failure to clear the acute infection and parasite-persistence-associated pathologic events, leading to cardiomyopathy and heart failure in CD.

Based on the above discussed literature, an effective vaccine, whether given prophylactically or therapeutically, is envisioned to: (1) target all parasite forms that circulate in the bloodstream and replicate or hide in tissues, (2) recognize conserved antigens of all clinically relevant parasite lineages and strains that are known to cause infection in mammals, and (3) induce a long-lasting memory immune response that can be recalled to rapidly control/eliminate *T. cruzi* infection.

## 3. Vaccine Development Against *T. cruzi* and Chagas Disease

Considering the complexity of the antigenic variability in *T. cruzi* populations that leads to diverse immune responses, the goal of achieving sterilizing immunity in which parasite infection and transmission is eliminated by a vaccine is noble and magnanimous but lacks consideration for those that have the least resources and would be maximally benefited by even a partially protective vaccine. Indeed, current efforts have led to the development of candidate vaccines that elicit sufficient immune memory to reduce the parasite load below a threshold level such that a vaccinated individual is protected from chronic Chagas disease. It is highly likely that identification and testing of several candidate vaccines by individual investigators will lead to the selection of a panel of key *T. cruzi* antigens that together will offer a multivalent, highly efficacious vaccine against Chagas disease.

Over the last century, several candidate vaccines have been tested in small animals with varying degree of success in controlling the *T. cruzi* infection and/or tissue damage. Early efforts utilized whole parasites killed by various methods or sub-cellular fractions of the parasite as a vaccine that generated a relevant immune response, evidenced by control of acute challenge infection and survival from lethal infection [41]. Parasite strains attenuated by repeat passage in culture (e.g., TCC) were also used as a vaccine and were shown to provide protection from subsequent challenge with a virulent strain of *Tc* in mice and dogs [42,43]. Researchers also promoted *Trypanosoma rangeli*, which shares significant sequence homology with *T. cruzi* but does not cause disease in humans, as a prophylactic vaccine [44,45]. However, our studies suggest that *T. rangeli* did not offer significant protection from *Tc* infection when used by itself and did not enhance the protective efficacy of a subunit DNA vaccine [46]. With the advancement of genetic engineering technology, investigators have focused on knocking down virulence genes to generate live *Tc* vaccines. Examples include the use of *Tc* deficient in calmodulin-ubiquitin (TulCub8), calreticulin, *Lyt1*, *gp72*, *dhfr-ts*, *Ech1/2*, and other genes as a vaccine, which offered immunity to parasitemia caused by wild-type strains in in-bred and out-bred mice and hamsters [41]. A major limitation of whole organism vaccines is the generation of large quantities of attenuated parasites, without a gain in virulence. Some researchers also remain concerned that an attenuated live vaccine may cause parasitemia and disease in immunocompromised individuals [47]. Overall, concerns about the use of live attenuated vaccines may outweigh the benefits offered for the control of Chagas disease.

### 3.1. Subunit Vaccine Candidates

Garg et al. utilized recombinant *T. cruzi* expressing a model antigen (chicken ovalbumin) in different cellular compartments to demonstrate that GPI (glycosylphosphatidylinositol)-anchored proteins expressed in the infective and intracellular stages of *T. cruzi* that are released in host cell cytoplasm during parasite differentiation are the most likely source of peptides for immune activation of B and T cells [48]. Equipped with this information, investigators focused on identifying the abundantly expressed surface antigens of the large families, e.g., trans-sialidases, mucin-associated surface proteins (MASP), and glycoprotein 63, as vaccine candidates. Indeed, members of the trans-sialidases superfamily (e.g., TSA1, ASP1/2, ASP9, TS) were recognized by the antibodies and CD8^+^T lymphocytes in infected mice and humans [49]. Other antigens, including complement regulatory protein (CRP), cruzipain, Tc24, GP82, KMP11, LYT1, paraflagellar rod proteins (PFR), and TC52, were also identified as potential vaccine candidates, because they were recognized by antibodies and IFNγ-producing CD8^+^T cells in experimental models of *Tc* infection and Chagas patients [50].

We performed a computational screening of the *T. cruzi* sequence database for candidate surface antigens and biological screening to select those that were recognized by IgGs and type 1 CD8^+^T cells in infected mice, dogs, or humans [51,52]. Of the 11 antigens thus selected, TcG1, TcG2, and TcG4 exhibited additional desirable features, as they were (a) expressed in both infective and intracellular forms of *T. cruzi*, (b) released into host cell cytoplasm during parasite differentiation, and (c) consisted of epitopes presented by the MHC alleles of mice, dogs, and humans [51,52]. Notably, TcG2 and TcG4 were conserved in five of the six *T. cruzi* lineages (80–96% homology) and thus relevant as vaccine candidates for the control of diverse *Tc* isolates circulating in the Americas. Others have employed subtractive proteomics for the selection of immunodominant epitopes and identified MASP1/2 as potential vaccine candidates [53].

### 3.2. Subunit Vaccines

Investigators have tested the prophylactic and therapeutic efficacy of single- and multi-component candidate vaccines in various experimental models. In general, DNA immunization has been favored due to the ease of production and DNA stability, and because antigen delivery by DNA vaccination was found to be efficient in eliciting antibodies, Th1 cytokines, and CD8^+^T cell responses to encoded antigens. In parallel studies, adjuvants (e.g., IL12, GMCSF, CD40, HSP70, CpG-ODN, c-di-AMP) were tested to enhance the Th1 responses to defined antigen vaccines.

Initial studies focused on evaluating the vaccine efficacy of antigens of the large families of surface proteins. Members of trans-sialidases family (e.g., ASP1, ASP2, TSA1, TS, SAPA, TSf) have been tested as a DNA or protein vaccine (individually or in combination) and offered a degree of immune resistance to *T. cruzi* infection that correlated with the amount and type of antigen delivered with and without adjuvants (reviewed in [49]). Yet, none of these experimental vaccines elicited sterile immunity, and they have not reached the clinical trial phase. No protection was observed in mice immunized with genes encoding members of the mucin family, though a KLH-conjugated synthetic peptide from the MASP family did offer survival benefits from lethal infection in mice [54]. It can be surmised that the shared epitopes expressed by members of the large families prevent robust activation of the immune system against *Tc*, and a combination of epitopes from other conserved genes would be more useful in stimulating immune effectors against diverse parasite strains.

Subsequently, investigators used a variety of delivery vehicles to test the vaccine efficacy of non-family proteins in experimental models. For example, oral delivery of *Salmonella*-carrying cruzipain DNA (SCz) induced mucosal IgA response, while boosting oral SCz with recombinant cruzipain protein and CpG-ODN produced a strong systemic immune response capable of controlling challenge infection and tissue damage [55]. Vaccines based on PFR2/3 induced IgG2a response; however, only PFR2 conjugated with HSP70 induced IL12 and IFNγ expression and a degree of protection from *Tc* infection [56,57]. Immunization with Tc24, Tcb3, and LYT1 induced cytotoxic T cells; however, only LYT1 provided resistance to lethal infection in mice [58], thus suggesting that antigens recognized by the host immune response after infection may not always be the best candidates for vaccine development. Adenovirus 5 was used as a delivery vehicle by Brazilian scientists; these studies showed that prophylactic Ad-ASP2/Ad-TS elicited robust type 1 cytokines (TNFα, IFNγ) producing CD8^+^ effector T cell response and 100% survival from lethal challenge infection in Balb/c and C57BL/6 mice [59,60]. The same group showed that therapeutic Ad-ASP2/Ad-TS reprogrammed the immune response such that parasite-persistence-associated oxidative/inflammatory stress, cardiac electrical alterations, and cardiac tissue damage were significantly reduced in chronically infected mice [61]. Ad-ASP2 itself was also found to enhance the inflammatory gene expression profile and decrease amastigote nests in the tissues of mice infected at the time of immunization [62]. Galα(1,3)Galβ(1,4)GlcNAc has been developed as a vaccine candidate in conjugation with human serum albumin with the aim of stimulating anti-α-Gal antibodies, which were shown to provide up to 99.9% protection from tissue parasites in α1,3-galactosyltransferase knockout mice, while non-vaccinated mice exhibited a high degree of tissue parasites, necrotic myocytes, and extensive cardiac inflammation [63].

We have evaluated the protective efficacy of TcG1, TcG2, and TcG4 in mice and dogs, delivering these antigens (individually or in combination) by homologous and heterologous prime/boost approaches (e.g., DNA/DNA, DNA/protein, DNA/Modified Vaccinia Ankara). In all cases, vaccine provided 80–95% protection from acute parasitemia and chronic myocarditis in mice [46,64,65,66]. The overall extent of protection was primarily associated with elicitation of Th1 cytokine response and CD8^+^T cell cytolytic activity, while trypanolytic antibodies were not induced by these vaccine candidates. Further studies showed that TcG1 was expendable, and only TcG2/TcG4-based nanovaccine that utilized an antibiotic-free plasmid for delivery was sufficient to offer effective control of infection and disease pathology after primary infection, as well as eliciting a potent recall response potentially capable of controlling the repeat infection [67]. Importantly, the TcG2- and TcG4-encoding nanovaccine-induced immune response to challenge infection subsided after parasite control, and inflammatory infiltrate and oxidative damage were decreased in the tissues of chronically infected, vaccinated mice [68]. As in mice, dogs immunized with the three candidate antigens (TcG1, TcG2, TcG4) also showed significant protection from challenge infection and acute myocarditis [69,70]. TcSP- and TcSSP4-encoding DNA vaccines have also been found to induce anti-parasite IgG2- and IFNγ-producing lymphocytic cell proliferation in dogs, leading to moderate control of tissue parasites and electrocardiographic aberrations [71].

Building on these achievements, in recent studies, investigators have developed chimeric antigens incorporating epitopes of multiple antigens in one vaccine. For example, trivalent cruzipain-TS-ASP2 vaccine (Traspain) showed promising protection from *Tc* infection in mice [72]. We have engineered a bivalent vaccine with or without a string of adjuvants that exhibited prophylactic and therapeutic efficacy, evidenced by >97% control of parasite and tissue damage (unpublished).

Congenital transmission of *T. cruzi* from infected mothers to their newborns has emerged as a major cause of Chagas disease in humans [73]. Experts agree that seropositive women of reproductive age should be treated with benznidazole to potentially prevent congenital transmission [74], and there are no available drugs for the treatment of infected mothers during pregnancy. However, there is only one published report testing the efficacy of a candidate vaccine in pregnant mice. Garg and coworkers found that *Tc* infection resulted in delayed pregnancy, and pregnant female mice gave birth to pups with lower survival rates, decreased birth weight, and slower growth [75]. TcG2- and TcG4-encoding nanovaccine was found to be safe during pregnancy, and it modulated the maternal and placental T cell immune response such that maternal and placental parasite burden was reduced, and vaccine improved the placental integrity and birth/survival rate of newborns of infected mothers [76].

So far, most investigators have devoted efforts to testing the efficacy of their selected candidate vaccines against 1–2 *T. cruzi* isolates or strains of interest in in-bred experimental mouse models that could be established in their laboratories. Considering the diverse geographic and genetic complexity of *T. cruzi* isolates of different lineages, the next phase of studies in vaccine development should address if the candidate vaccine is effective against multiple *T. cruzi* isolates of clinical importance in out-bred animal models or naturally infected animals. It is hoped that candidate antigens that are highly conserved among different parasite lineages and expressed in all life cycle stages of the parasite will be successful in offering broad-range immunity to *T. cruzi* infection.

### 3.3. Therapeutic Vaccines Against CD

The overall basis for therapeutic vaccines is to enhance multiple immune effector mechanisms to prevent/reduce the parasite’s persistence. A therapeutic DNA vaccine encoding Tc52, TSA1, and Tc24 enhanced the number of CD4^+^ and CD8^+^ T cells and decreased the parasitemia and mortality in acutely infected mice [77] but failed to arrest cardiomyopathy in chronically infected dogs [78]. Others showed that ASP2- and TS-encoding therapeutic DNA vaccine was not effective in limiting parasitemia or mortality [79] and TSA1 DNA vaccine enhanced myocarditis [80] in infected mice. Subsequently, Tc24 protein was encapsulated in poly(lactic-co-glycolic acid) nanoparticles (with and without E6020 adjuvant), and this vaccine was found to enhance the splenic type 1 CD8^+^T cell response, such that tissue parasites were reduced when given to mice during acute infection with a highly lethal *T. cruzi* H1 strain, leading to parasite clearance in 60% of chronically infected mice [81,82]. At least one report has recently documented the safety and efficacy of TSA1/Tc24-based therapeutic DNA vaccine in controlling cardiac alterations in a small group of experimentally infected macaques [83].

Considering the limited success of therapeutic vaccines, and the fact that repeat challenge with *Tc* continued to boost the immune responses but did not provide protection from cardiac damage in mice [84], it is proposed that immune therapy against parasite persistence should be adjuvanted with other agents to prevent cardiac damage. Towards this goal, it was found that treatment with benznidazole during the indeterminate phase (when most seropositive patients are identified) controlled the parasite persistence, but did not avert cardiac remodeling and deterioration of ventricular contractility in infected mice and rats. When an antioxidant (phenyl-alpha-tert-butyl nitrone) was added to a benznidazole treatment regimen, mitochondrial function and left ventricular contractile activity were improved in Chagas hearts [85,86]. Likewise, a therapeutic TcG2/TcG4-based DNA vaccine offered better control of the oxidative damage caused by mitochondrial deficiencies of electron transport chain in glutathione-peroxidase-overexpressing mice, while progressive cardiac damage was noted in wildtype mice after TG2/TcG4 therapeutic vaccine [87]. Thus, it is safe to surmise that therapeutic vaccines designed to achieve a rapid stimulation of humoral and cytotoxic immunity to attack persistent parasites, along with adjunct therapies capable of controlling the onset of oxidative insult and mitochondrial deficiencies, would prove to be maximally beneficial in preserving cardiac structure and function in Chagas disease.

## 4. Future Opportunities and Challenges

Research efforts have led to the identification of several candidate antigens that exhibit promising prophylactic and therapeutic efficacy as a single-component, multi-component, or chimeric vaccine in experimental models of *T. cruzi* infection and Chagas disease. None of the current vaccines offer sterilizing immunity and efforts toward improving vaccine immunogenicity have continued. A recent presentation by Dr. SMR Teixiera and colleagues at the 2024 Brazilian Society of Protozoology meeting deserves mention here: The authors found that TcTS with trans-sialidase enzymatic activity is highly immunogenic but offers low protective efficacy as a vaccine candidate. Further studies using TS deletion mutants led to the findings that the C-terminal repeats known as SAPA (shed acute phase antigen) domain serve as a negative modulator of TS-induced immune response and instead offer virulence to the parasite. Armed with this knowledge, the authors are testing the efficacy of SAPA-deficient TS as mRNA and protein vaccines encapsulated in lipid nanoparticles, and hope that this novel vaccine will be highly protective against Chagas disease. Others reported that incorporation of low-dose benznidazole enhanced the therapeutic efficacy of a bivalent recombinant protein vaccine (TSA1/Tc24) in controlling cardiac fibrosis in chronically infected mice [88]. This study is particularly important because benznidazole itself exhibits lower effectiveness in the chronic phase, likely due to its limited reach to the niches where the parasite hides or its lower effectiveness against the dormant state parasite [89]. Thus, anti-parasite drugs (both old and new) adjuvanted with a vaccine offer a promising approach for controlling Chagas disease.

Development of the next generation of vaccines will likely involve efforts focused on enhancing the in vivo stability of vaccines, such that prolonged antigen presentation and immune memory is achieved. For example, delivery of DNA vaccines encapsulated in cationic liposomes [90] and biopolymers [91], which protect DNA from nucleases, could enhance the stability and expression of antigens carried by DNA vaccines. Liposomes have also been utilized to co-deliver adjuvant such as poly I:C RNA or CpG-ODN to enhance the immunogenicity of DNA and mRNA vaccines [90] and can be designed to carry such adjuvants for DNA vaccines against *T. cruzi*. Other platforms for immunogenic vaccine delivery have also been developed. For example, the assembly of candidate antigens in virus-like particles (VLPs) could enhance antigen presentation and innate and adaptive immunity [92]. T4 bacteriophage can also provide an excellent platform for generating nanoparticle subunit vaccine [93]. The technological knowledge gained from the development of VLP- and T4-phage-based vaccines for high-risk viruses and bacteria could potentially be employed for generating such vaccines for pathogenic parasites. Oral vaccines using tobacco plant for antigen expression exhibited immunologic protection from malaria, toxoplasmosis, and other parasitic diseases [94], and offer a promising perspective for developing cost-effective plant-based vaccines to continue the fight against Chagas disease. Testing the safety and efficacy of candidate vaccines during pregnancy, with the aim of decreasing the impact of congenital Chagas disease, is also urgently needed.

Investigators also need to ensure that the subunit vaccine (along with the delivery vehicle) is safe, elicits recall immune response to protect against repeat infection from the same or different clinically relevant parasite isolates, and is efficacious in reducing the cardiac damage caused by chronic infection in multiple hosts (e.g., mice, dogs, non-human primates). Vaccines that can be delivered orally or via skin patches and stored long-term at room temperature will likely have a better chance of reaching the marketplace. Alternatively, one can envision incorporating *Tc*-specific antigenic epitopes into vaccines for SARS-CoV2 or other high-risk infectious agents to facilitate the vaccination against Chagas disease.

Demonstrating the long-term effectiveness of the current and new anti-parasite vaccines and drugs against a variety of parasite strains of different lineages remains a serious challenge. Such studies are extremely expensive and cannot be carried out with limited availability of resources. Furthermore, *T. cruzi* has a large, diploid genome, in which coding sequence may undergo recombination events to further contribute to antigenic complexity, challenging the development of new vaccines and therapies.

A general challenge for testing the prophylactic efficacy of candidate vaccines in humans is the low incidence rate of disease development over decades. Clinical studies testing the therapeutic efficacy of candidate vaccines in already infected individuals are more feasible; however, the recruitment and long-term follow up required to monitor parasite clearance, immunity, and protection from chronic cardiomyopathy remains a major obstacle. The response to treatment in an infected individual is measured by achievement of negative serology, which can take decades. Some investigators have conducted seminal studies identifying new surrogate markers that can offer a robust readout of vaccine efficacy in the short term and potentially be used in clinical trials testing new drugs and vaccines against CD [95,96].

Finally, it is hoped that the most advanced drugs and vaccines will receive strong support from the academic–private–government partnership and overcome the barriers of costs associated with vaccine testing and production for a neglected tropical disease. Joint collaborative efforts will lead to the development of vaccines that address the antigenic variance of diverse *T. cruzi* lineages circulating in different parts of the Americas, elicit broad and long-lasting immunity to all clinically relevant parasite strains, and offer protection from infection. While testing and developing a vaccine for humans, a vaccine for veterinary use would offer a promising approach to prevent parasite transmission in domestic reservoirs and indirectly protect humans from *T. cruzi* infection.

## Data Availability

No new data were created or analyzed in this study.

## References

[1] Bonney K.M., Luthringer D.J., Kim S.A., Garg N.J., Engman D.M. (2020). Pathology and pathogenesis of chagas heart disease. Annu. Rev. Pathol. Mech. Dis..

[2] World Health Organization (2010). Chagas Disease: Control and Elimination.

[3] Limon-Flores A.Y., Cervera-Cetina R., Tzec-Arjona J.L., Ek-Macias L., Sanchez-Burgos G., Ramirez-Sierra M.J., Cruz-Chan J.V., VanWynsberghe N.R., Dumonteil E. (2010). Effect of a combination DNA vaccine for the prevention and therapy of *Trypanosoma cruzi* infection in mice: Role of CD4^+^ and CD8^+^ T cells. Vaccine.

[4] Moncayo A., Silveira A.C. (2009). Current epidemiological trends for Chagas disease in Latin America and future challenges in epidemiology, surveillance and health policy. Mem. Inst. Oswaldo Cruz..

[5] Lee B.Y., Bacon K.M., Bottazzi M.E., Hotez P.J. (2013). Global economic burden of Chagas disease: A computational simulation model. Lancet Infect. Dis..

[6] Lee B.Y., Bacon K.M., Connor D.L., Willig A.M., Bailey R.R. (2010). The potential economic value of a *Trypanosoma cruzi* (Chagas disease) vaccine in Latin America. PLoS Negl. Trop. Dis..

[7] Gutierrez F.R., Guedes P.M., Gazzinelli R.T., Silva J.S. (2009). The role of parasite persistence in pathogenesis of Chagas heart disease. Parasite Immunol..

[8] Vellozo N.S., Matos-Silva T.C., Lopes M.F. (2023). Immunopathogenesis in *Trypanosoma cruzi* infection: A role for suppressed macrophages and apoptotic cells. Front. Immunol..

[9] Lewis M.D., Francisco A.F., Jayawardhana S., Langston H., Taylor M.C., Kelly J.M. (2018). Imaging the development of chronic Chagas disease after oral transmission. Sci. Rep..

[10] Combs T.P., Nagajyothi, Mukherjee S., de Almeida C.J., Jelicks L.A., Schubert W., Lin Y., Jayabalan D.S., Zhao D., Braunstein V.L. (2005). The adipocyte as an important target cell for *Trypanosoma cruzi* infection. J. Biol. Chem..

[11] Ferreira A.V., Segatto M., Menezes Z., Macedo A.M., Gelape C., de Oliveira Andrade L., Nagajyothi F., Scherer P.E., Teixeira M.M., Tanowitz H.B. (2011). Evidence for *Trypanosoma cruzi* in adipose tissue in human chronic chagas disease. Microbes Infect..

[12] Sanchez-Valdez F.J., Padilla A., Wang W., Orr D., Tarleton R.L. (2018). Spontaneous dormancy protects *Trypanosoma cruzi* during extended drug exposure. Elife.

[13] Marin-Neto J.A., Cunha-Neto E., Maciel B.C., Simoes M.V. (2007). Pathogenesis of chronic Chagas heart disease. Circulation.

[14] Choudhuri S., Rios L., Vazquez-Chagoyan J.C., Garg N.J. (2021). Oxidative stress implications for therapeutic vaccine development against Chagas disease. Expert. Rev. Vaccines.

[15] Maldonado E., Rojas D.A., Urbina F., Solari A. (2021). The oxidative stress and chronic inflammatory process in Chagas disease: Role of exosomes and contributing genetic factors. Oxid. Med. Cell. Longev..

[16] de Oliveira T.B., Pedrosa R.C., Filho D.W. (2007). Oxidative stress in chronic cardiopathy associated with Chagas disease. Int. J. Cardiol..

[17] Lopez M., Tanowitz H.B., Garg N.J. (2018). Pathogenesis of chronic Chagas disease: Macrophages, mitochondria, and oxidative stress. Curr. Clin. Microbiol. Rep..

[18] de Carvalho F.C.T., de Oliveira L.R.C., Gatto M., Tasca K.I., da Silva L.D.M., dos Santos K.C., Pierine D.T., da Costa E.A.P.N., Francisqueti-Ferron F.V., dos Santos R.M. (2023). Oxidative stress evaluation in patients with chronic Chagas disease. Parasitol. Int..

[19] Sanchez-Villamil J.P., Bautista-Nino P.K., Serrano N.C., Rincon M.Y., Garg N.J. (2020). Potential role of antioxidants as adjunctive therapy in Chagas disease. Oxid. Med. Cell. Longev..

[20] Wan X., Garg N.J. (2021). Sirtuin control of mitochondrial dysfunction, oxidative stress, and inflammation in Chagas disease models. Front. Cell Infect. Microbiol..

[21] Choudhuri S., Chowdhury I.H., Garg N.J. (2020). Mitochondrial regulation of macrophage response against pathogens. Front. Immunol..

[22] Rios L., Campos E.E., Menon R., Zago M.P., Garg N.J. (2020). Epidemiology and pathogenesis of maternal-fetal transmission of *Trypanosoma cruzi* and a case for vaccine development against congenital chagas disease. Biochim. Biophys. Acta Mol. Basis Dis..

[23] Maldonado E., Morales-Pison S., Urbina F., Solari A. (2022). Vaccine design against Chagas disease focused on the use of nucleic acids. Vaccines.

[24] Pinazo M.J., Malchiodi E., Ioset J.R., Bivona A., Gollob K.J., Dutra W.O. (2024). Challenges and advancements in the development of vaccines and therapies against Chagas disease. Lancet Microbe.

[25] Farani P.S.G., Jones K.M., Poveda C. (2024). Treatments and the perspectives of developing a vaccine for Chagas disease. Vaccines.

[26] Ramírez-Toloza G., Ferreira A. (2017). *Trypanosoma cruzi* evades the complement system as an efficient strategy to survive in the mammalian host: The specific roles of host/parasite molecules and trypanosoma cruzi calreticulin. Front. Microbiol..

[27] Almeida I.C., Camargo M.M., Procopio D.O., Silva L.S., Mehlert A., Travassos L.R., Gazzinelli R.T., Ferguson M.A. (2000). Highly purified glycosylphosphatidylinositols from *Trypanosoma cruzi* are potent proinflammatory agents. EMBO J..

[28] Tanowitz H.B., Wen J.J., Machado F.S., Desruisseaux M.S., Robello C., Garg N.J., Gavins F.N.E., Stokes K.Y. (2016). Trypanosoma cruzi and Chagas disease: Innate immunity, ROS, and cardiovascular system. Vascular Responses to Pathogens.

[29] Cronemberger-Andrade A., Xander P., Soares R.P., Pessoa N.L., Campos M.A., Ellis C.C., Grajeda B., Ofir-Birin Y., Almeida I.C., Regev-Rudzki N. (2020). *Trypanosoma cruzi*-infected human macrophages shed proinflammatory extracellular vesicles that enhance host-cell invasion via toll-like receptor 2. Front. Cell Infect. Microbiol..

[30] Koo S.J., Szczesny B., Wan X., Putluri N., Garg N.J. (2018). Pentose phosphate shunt modulates reactive oxygen species and nitric oxide production controlling *Trypanosoma cruzi* in macrophages. Front. Immunol..

[31] Koo S.J., Garg N.J. (2019). Metabolic programming of macrophage functions and pathogens control. Redox Biol..

[32] Cerbán F.M., Stempin C.C., Volpini X., Carrera Silva E.A., Gea S., Motran C.C. (2020). Signaling pathways that regulate *Trypanosoma cruzi* infection and immune response. Biochim. Biophys. Acta-Mol. Basis Dis..

[33] Macaluso G., Grippi F., Di Bella S., Blanda V., Gucciardi F., Torina A., Guercio A., Cannella V. (2023). A review on the immunological response against *Trypanosoma cruzi*. Pathogens.

[34] Rodrigues M.M., Ribeirão M., Boscardin S.B. (2000). CD4^+^ Th1 but not Th2 clones efficiently activate macrophages to eliminate *Trypanosoma cruzi* through a nitric oxide dependent mechanism. Immunol. Lett..

[35] Hoft D.F., Schnapp A.R., Eickhoff C.S., Roodman S.T. (2000). Involvement of CD4^+^ Rh1 cells in systemic immunity protective against primary and secondary challenges with *Trypanosoma cruzi*. Infect. Immun..

[36] Hodžić A., Mateos-Hernández L., de la Fuente J., Cabezas-Cruz A. (2020). A-gal-based vaccines: Advances, opportunities, and perspectives. Trends Parasitol..

[37] Acosta Rodríguez E.V., Araujo Furlan C.L., Fiocca Vernengo F., Montes C.L., Gruppi A. (2019). Understanding CD8^+^T cell immunity to *Trypanosoma cruzi* and how to improve it. Trends Parasitol..

[38] Pérez-Antón E., Egui A., Thomas M.C., Simón M., Segovia M., López M.C. (2020). Immunological exhaustion and functional profile of CD8^+^T lymphocytes as cellular biomarkers of therapeutic efficacy in chronic Chagas disease patients. Acta Trop..

[39] Dutra W.O., Menezes C.A., Magalhaes L.M., Gollob K.J. (2014). Immunoregulatory networks in human Chagas disease. Parasite Immunol..

[40] Medina-Rincón G.J., Gallo-Bernal S., Jiménez P.A., Cruz-Saavedra L., Ramírez J.D., Rodríguez M.J., Medina-Mur R., Díaz-Nassif G., Valderrama-Achury M.D., Medina H.M. (2021). Molecular and clinical aspects of chronic manifestations in Chagas disease: A state-of-the-art review. Pathogens.

[41] Solana J.C., Moreno J., Iborra S., Soto M., Requena J.M. (2022). Live attenuated vaccines, a favorable strategy to provide long-term immunity against protozoan diseases. Trends Parasitol..

[42] Revelli S., Basombrio M.A., Valenti J.L., Moreno H., Poli H., Morini J.C. (1993). Evaluation of an attenuated *Trypanosoma cruzi* strain in rats. Analysis of survival, parasitemia and tissue damage. Medicina.

[43] Basombrio M.A., Segura M.A., Mora M.C., Gomez L. (1993). Field trial of vaccination against American Trypanosomiasis (Chagas’ disease) in dogs. Am. J. Trop. Med. Hyg..

[44] Basso B., Marini V. (2015). Experimental Chagas disease in balb/c mice previously vaccinated with *T. rangeli*. II. The innate immune response shows immunological memory: Reality or fiction?. Immunobiol..

[45] Basso B., Moretti E., Fretes R. (2014). Vaccination with *Trypanosoma rangeli* induces resistance of guinea pigs to virulent *Trypanos Cruzi*. Vet. Immunol. Immunopathol..

[46] Gupta S., Salgado-Jimenez B., Lokugamage N., Vazquez-Chagoyan J.C., Garg N.J. (2019). Tcg2/Tcg4 DNA vaccine induces th1 immunity against acute *Trypanosoma cruzi* infection: Adjuvant and antigenic effects of heterologous *T. rangeli* booster immunization. Front. Immunol..

[47] Sanchez-Valdez F.J., Perez Brandan C., Ferreira A., Basombrio M.A. (2015). Gene-deleted live-attenuated *Trypanosoma cruzi* parasites as vaccines to protect against Chagas disease. Expert. Rev. Vaccines.

[48] Garg N.J., Nunes M.P., Tarleton R.L. (1997). Delivery by *Trypanosoma cruzi* of proteins into the MHC class I antigen processing and presentation pathway. J. Immunol..

[49] da Costa K.M., Marques da Fonseca L., Dos Reis J.S., Santos M., Previato J.O., Mendonca-Previato L., Freire-de-Lima L. (2021). *Trypanosoma cruzi* trans-sialidase as a potential vaccine target against Chagas disease. Front. Cell Infect. Microbiol..

[50] Rios L.E., Vazquez-Chagoyan J.C., Pacheco A.O., Zago M.P., Garg N.J. (2019). Immunity and vaccine development efforts against *Trypanosoma cruzi*. Acta Trop..

[51] Bhatia V., Sinha M., Luxon B., Garg N.J. (2004). Utility of *Trypanosoma cruzi* sequence database for the identification of potential vaccine candidates: In silico and in vitro screening. Infect. Immun..

[52] Bhatia V., Garg N.J. (2008). Previously unrecognized vaccine candidates control *Trypanosoma cruzi* infection and immunopathology in mice. Clin. Vaccine Immunol..

[53] Albaqami F.F., Altharawi A., Althurwi H.N., Alharthy K.M. (2024). From proteome to candidate vaccines: Target discovery and molecular dynamics-guided multi-epitope vaccine engineering against kissing bug. Front. Immunol..

[54] Serna C., Lara J.A., Rodrigues S.P., Marques A.F., Almeida I.C., Maldonado R.A. (2014). A synthetic peptide from *Trypanosoma cruzi* mucin-like associated surface protein as candidate for a vaccine against Chagas disease. Vaccine.

[55] Cazorla S.I., Becker P.D., Frank F.M., Ebensen T., Sartori M.J., Corral R.S., Malchiodi E.L., Guzman C.A. (2008). Oral vaccination with *Salmonella enterica* as a cruzipain-DNA delivery system confers protective immunity against *Trypanosoma cruzi*. Infect. Immun..

[56] Morell M., Thomas M.C., Caballero T., Alonso C., Lopez M.C. (2006). The genetic immunization with paraflagellar rod protein-2 fused to the hsp70 confers protection against late *Trypanosoma cruzi* infection. Vaccine.

[57] Egui A., Thomas M.C., Morell M., Maranon C., Carrilero B., Segovia M., Puerta C.J., Pinazo M.J., Rosas F., Gascon J. (2012). *Trypanosoma cruzi* paraflagellar rod proteins 2 and 3 contain immunodominant CD8^+^T-cell epitopes that are recognized by cytotoxic t cells from chagas disease patients. Mol. Immunol..

[58] Fralish B.H., Tarleton R.L. (2003). Genetic immunization with Lyt1 or a pool of trans-sialidase genes protects mice from lethal *Trypanosoma cruzi* infection. Vaccine.

[59] Vasconcelos J.R., Hiyane M.I., Marinho C.R., Claser C., Machado A.M., Gazzinelli R.T., Bruna-Romero O., Alvarez J.M., Boscardin S.B., Rodrigues M.M. (2004). Protective immunity against *Trypanosoma cruzi* infection in a highly susceptible mouse strain after vaccination with genes encoding the amastigote surface protein-2 and trans-sialidase. Hum. Gene Ther..

[60] Vasconcelos J.R., Dominguez M.R., Neves R.L., Ersching J., Araujo A., Santos L.I., Virgilio F.S., Machado A.V., Bruna-Romero O., Gazzinelli R.T. (2014). Adenovirus vector-induced CD8^+^T effector memory cell differentiation and recirculation, but not proliferation, are important for protective immunity against experimental *Trypanosoma cruzi* infection. Hum. Gene Ther..

[61] Pereira I.R., Vilar-Pereira G., Marques V., da Silva A.A., Caetano B., Moreira O.C., Machado A.V., Bruna-Romero O., Rodrigues M.M., Gazzinelli R.T. (2015). A human type 5 adenovirus-based *Trypanosoma cruzi* therapeutic vaccine re-programs immune response and reverses chronic cardiomyopathy. PLoS Pathog..

[62] Ribeiro F.A.P., Pontes C., Machado A.M.V., Bruna-Romero O., Quintana H.T., De Oliveira F., De Vasconcelos J.R.C., Ribeiro D. (2019). A Therapeutical effects of vaccine from *Trypanosoma cruzi* amastigote surface protein 2 by simultaneous inoculation with live parasites. J. Cell Biochem..

[63] Portillo S., Zepeda B.G., Iniguez E., Olivas J.J., Karimi N.H., Moreira O.C., Marques A.F., Michael K., Maldonado R.A., Almeida I.C. (2019). A prophylactic α-gal-based glycovaccine effectively protects against murine acute chagas disease. NPJ Vaccines.

[64] Gupta S., Garg N.J. (2010). Prophylactic efficacy of TcVac2 against *Trypanosoma cruzi* in mice. PLoS Negl. Trop. Dis..

[65] Gupta S., Garg N.J. (2013). TcVac3 induced control of *Trypanosoma cruzi* infection and chronic myocarditis in mice. PLOS ONE.

[66] Gupta S., Garg N.J. (2015). A two-component DNA-prime/protein-boost vaccination strategy for eliciting long-term, protective T cell immunity against *Trypanosoma cruzi*. PLoS Pathog..

[67] Chowdhury I.H., Lokugamage N., Garg N.J. (2020). Experimental nanovaccine offers protection against repeat exposures to *Trypanosoma cruzi* through activation of polyfunctional T cell response. Front. Immunol..

[68] Lokugamage N., Choudhuri S., Davies C., Chowdhury I.H., Garg N.J. (2020). Antigen-based nano-immunotherapy controls parasite persistence, inflammatory and oxidative stress, and cardiac fibrosis, the hallmarks of chronic Chagas cardiomyopathy, in a mouse model of *Trypanosoma cruzi* infection. Vaccines.

[69] Aparicio-Burgos J.E., Ochoa-Garcia L., Zepeda-Escobar J.A., Gupta S., Dhiman M., Martinez J.S., de Oca-Jimenez R.M., Val Arreola M., Barbabosa-Pliego A., Vazquez-Chagoyan J.C. (2011). Testing the efficacy of a multi-component DNA-prime/DNA-boost vaccine against *Trypanosoma cruzi* infection in dogs. PLoS Negl. Trop. Dis..

[70] Aparicio-Burgos J.E., Zepeda-Escobar J.A., de Oca-Jimenez R.M., Estrada-Franco J.G., Barbabosa-Pliego A., Ochoa-Garcia L., Alejandre-Aguilar R., Rivas N., Penuelas-Rivas G., Val-Arreola M. (2015). Immune protection against *Trypanosoma cruzi* induced by TcVac4 in a canine model. PLoS Negl. Trop. Dis..

[71] Arce-Fonseca M., Ballinas-Verdugo M.A., Zenteno E.R., Suarez-Flores D., Carrillo-Sanchez S.C., Alejandre-Aguilar R., Rosales-Encina J.L., Reyes P.A., Rodriguez-Morales O. (2013). Specific humoral and cellular immunity induced by *Trypanosoma cruzi* DNA immunization in a canine model. Vet. Res..

[72] Sanchez Alberti A., Bivona A.E., Cerny N., Schulze K., Weissmann S., Ebensen T., Morales C., Padilla A.M., Cazorla S.I., Tarleton R.L. (2017). Engineered trivalent immunogen adjuvanted with a sting agonist confers protection against *Trypanosoma cruzi* infection. NPJ Vaccines.

[73] Chancey R.J., Edwards M.S., Montgomery S.P. (2023). Congenital Chagas disease. Pediatr. Rev..

[74] Alvarez M.G., Vigliano C., Lococo B., Bertocchi G., Viotti R. (2017). Prevention of congenital Chagas disease by benznidazole treatment in reproductive-age women. An observational study. Acta Trop..

[75] Rios L., Lokugamage N., Garg N.J. (2023). Effects of acute and chronic *Trypanosoma cruzi* infection on pregnancy outcomes in mice: Parasite transmission, mortality, delayed growth, and organ damage in pups. Am. J. Pathol..

[76] Rios L.E., Lokugamage N., Choudhuri S., Chowdhury I.H., Garg N.J. (2023). Subunit nanovaccine elicited T cell functional activation controls *Trypanosoma cruzi* mediated maternal and placental tissue damage and improves pregnancy outcomes in mice. NPJ Vaccines.

[77] Dumonteil E., Escobedo-Ortegon J., Reyes-Rodriguez N., Arjona-Torres A., Ramirez-Sierra M.J. (2004). Immunotherapy of trypanosoma cruzi infection with DNA vaccines in mice. Infect. Immun..

[78] Quijano-Hernandez I.A., Bolio-Gonzalez M.E., Rodriguez-Buenfil J.C., Ramirez-Sierra M.J., Dumonteil E. (2008). Therapeutic DNA vaccine against trypanosoma cruzi infection in dogs. Ann. N. Y. Acad. Sci..

[79] Sanchez-Burgos G., Mezquita-Vega R.G., Escobedo-Ortegon J., Ramirez-Sierra M.J., Arjona-Torres A., Ouaissi A., Rodrigues M.M., Dumonteil E. (2007). Comparative evaluation of therapeutic DNA vaccines against trypanosoma cruzi in mice. FEMS Immunol. Med. Microbiol..

[80] Zapata-Estrella H., Hummel-Newell C., Sanchez-Burgos G., Escobedo-Ortegon J., Ramirez-Sierra M.J., Arjona-Torres A., Dumonteil E. (2006). Control of trypanosoma cruzi infection and changes in t-cell populations induced by a therapeutic DNA vaccine in mice. Immunol. Lett..

[81] Barry M.A., Wang Q., Jones K.M., Heffernan M.J., Buhaya M.H., Beaumier C.M., Keegan B.P., Zhan B., Dumonteil E., Bottazzi M.E. (2016). A therapeutic nanoparticle vaccine against trypanosoma cruzi in a balb/c mouse model of chagas disease. Hum. Vaccin. Immunother..

[82] Barry M.A., Versteeg L., Wang Q., Pollet J., Zhan B., Gusovsky F., Bottazzi M.E., Hotez P.J., Jones K.M. (2019). A therapeutic vaccine prototype induces protective immunity and reduces cardiac fibrosis in a mouse model of chronic trypanosoma cruzi infection. PLoS Negl. Trop. Dis..

[83] Dumonteil E., Herrera C., Marx P.A. (2023). Safety and preservation of cardiac function following therapeutic vaccination against trypanosoma cruzi in rhesus macaques. J. Microbiol. Immunol. Infect..

[84] Rosas-Jorquera Christian E., Sardinha Luiz R., Pretel Fernando D., Bombeiro André L., D'Império Lima Maria R., Alvarez José M. (2013). Challenge of chronically infected mice with homologous trypanosoma cruzi parasites enhances the immune response but does not modify cardiopathy: Implications for the design of a therapeutic vaccine. Clin. Vaccine Immunol..

[85] Wen J.J., Bhatia V., Popov V.L., Garg N.J. (2006). Phenyl-alpha-tert-butyl nitrone reverses mitochondrial decay in acute chagas' disease. Am. J. Pathol..

[86] Wen J.J., Bhatia V., Popov V.L., Garg N.J. (2010). Phenyl-alpha-tert-butyl-nitrone and benzonidazole treatment controlled the mitochondrial oxidative stress and evolution of cardiomyopathy in chronic chagasic rats. J. Am. Coll. Cardiol..

[87] Gupta S., Smith C., Auclair S., Delgadillo Ade J., Garg N.J. (2015). Therapeutic efficacy of a subunit vaccine in controlling chronic *Trypanosoma cruzi* infection and chagas disease is enhanced by glutathione peroxidase over-expression. PLoS ONE.

[88] Dzul-Huchim V.M., Ramirez-Sierra M.J., Martinez-Vega P.P., Rosado-Vallado M.E., Arana-Argaez V.E., Ortega-Lopez J., Gusovsky F., Dumonteil E., Cruz-Chan J.V., Hotez P. (2022). Vaccine-linked chemotherapy with a low dose of benznidazole plus a bivalent recombinant protein vaccine prevents the development of cardiac fibrosis caused by *Trypanosoma cruzi* in chronically-infected balb/c mice. PLoS Negl. Trop. Dis..

[89] Barrett M.P., Kyle D.E., Sibley L.D., Radke J.B., Tarleton R.L. (2019). Protozoan persister-like cells and drug treatment failure. Nat. Rev. Microbiol..

[90] Schwendener R.A. (2014). Liposomes as vaccine delivery systems: A review of the recent advances. Ther. Adv. Vaccines.

[91] Franck C.O., Fanslau L., Bistrovic Popov A., Tyagi P., Fruk L. (2021). Biopolymer-based carriers for DNA vaccine design. Angew. Chem. Int. Ed. Engl..

[92] Kheirvari M., Liu H., Tumban E. (2023). Virus-like particle vaccines and platforms for vaccine development. Viruses.

[93] Tao P., Li Q., Shivachandra S.B., Rao V.B. (2017). Bacteriophage t4 as a nanoparticle platform to display and deliver pathogen antigens: Construction of an effective anthrax vaccine. Methods Mol. Biol..

[94] Ramos-Vega A., Monreal-Escalante E., Dumonteil E., Banuelos-Hernandez B., Angulo C. (2021). Plant-made vaccines against parasites: Bioinspired perspectives to fight against chagas disease. Expert. Rev. Vaccines.

[95] Cortes-Serra N., Losada-Galvan I., Pinazo M.J., Fernandez-Becerra C., Gascon J., Alonso-Padilla J. (2020). State-of-the-art in host-derived biomarkers of Chagas disease prognosis and early evaluation of anti-*Trypanosoma cruzi* treatment response. Biochim. Biophys. Acta-Mol. Basis Dis..

[96] Choudhuri S., Bhavnani S.K., Zhang W., Botelli V., Barrientos N., Iñiguez F., Zago M.P., Garg N.J. (2021). Prognostic performance of peripheral blood biomarkers in identifying seropositive individuals at risk of developing clinically symptomatic Chagas cardiomyopathy. Microbiol. Spectr..

